# PROSTATA: a framework for protein stability assessment using transformers

**DOI:** 10.1093/bioinformatics/btad671

**Published:** 2023-11-03

**Authors:** Dmitriy Umerenkov, Fedor Nikolaev, Tatiana I Shashkova, Pavel V Strashnov, Maria Sindeeva, Andrey Shevtsov, Nikita V Ivanisenko, Olga L Kardymon

**Affiliations:** Sber AI Lab, Moscow 105064, Russia; Bioinformatics Group, AIRI, Moscow 121170, Russia; Bioinformatics Group, AIRI, Moscow 121170, Russia; Bioinformatics Group, AIRI, Moscow 121170, Russia; Department of Computer Design and Technology, Bauman Moscow State Technical University, Moscow 105005, Russia; Bioinformatics Group, AIRI, Moscow 121170, Russia; Bioinformatics Group, AIRI, Moscow 121170, Russia; Regulatory Transcriptomics and Epigenomics Group, Institute of Bioengineering, Research Center of Biotechnology RAS, Moscow 117036, Russia; Bioinformatics Group, AIRI, Moscow 121170, Russia; Laboratory of Computational Proteomics, Institute of Cytology and Genetics SB RAS, Novosibirsk 630090, Russia; Bioinformatics Group, AIRI, Moscow 121170, Russia

## Abstract

**Motivation:**

Accurate prediction of change in protein stability due to point mutations is an attractive goal that remains unachieved. Despite the high interest in this area, little consideration has been given to the transformer architecture, which is dominant in many fields of machine learning.

**Results:**

In this work, we introduce PROSTATA, a predictive model built in a knowledge-transfer fashion on a new curated dataset. PROSTATA demonstrates advantage over existing solutions based on neural networks. We show that the large improvement margin is due to both the architecture of the model and the quality of the new training dataset. This work opens up opportunities to develop new lightweight and accurate models for protein stability assessment.

**Availability and implementation:**

PROSTATA is available at https://github.com/AIRI-Institute/PROSTATA and https://prostata.airi.net.

## 1 Introduction

Quantitative prediction of the effects of single amino acid substitutions on protein stability is a major problem that remains unresolved. Protein stability is related to its structure, function, and molecular evolution. The prediction of protein stability is part of a broader issue of predicting evolutionary fitness and the phenotypic effects of genomic variations.

**Figure 1.**
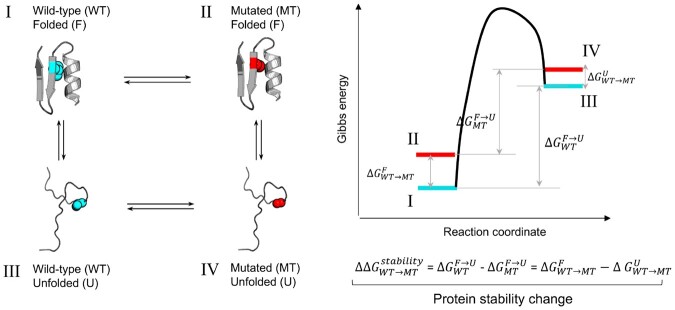
The difference in Gibbs free energy between the folded (F) and the ensemble of unfolded (U) states determines the protein stability. The effect of amino acid substitution on protein stability can be derived from the thermodynamic cycle (left). The free energy diagram illustrates the effect of amino acid substitution on the energy levels of folded and unfolded protein states (right). The wild-type (WT) and mutated (MT) amino acid residues are depicted as spheres.

Accurate predictions of changes in protein stability caused by mutations provide crucial insight into how proteins fold and function and also have important applications in the bioindustry. Amino acid substitutions in protein sequences can be stabilizing, destabilizing, or neutral, depending on whether the folded or unfolded states are favored compared to wild-type protein, or there is no effect (Fig. 1). The application of machine learning approaches capable of implicitly capturing changes in both states is particularly attractive.

Machine learning has irreversibly changed the landscape of computational biology and molecular modeling over the last few years. A plethora of tools designed to predict protein stability perfectly illustrate this change (Horne and Shukla 2022). We can roughly divide all the tools into three categories: (i) structural modeling methods employing some empirical energy function, (ii) ‘simple’ machine learning tools based on such methods as support vector machines (SVM), and (iii) deep neural networks, mostly convolutional neural networks (CNNs). The first category includes classical methods such as Rosetta (Kellogg *et al.* 2011, Alford *et al.* 2017, Leman *et al.* 2020), as well as newly developed methods, e.g. PoPMuSiC (Dehouck *et al.* 2011). Rosetta is a suite of macromolecular modeling programs (Kellogg *et al.* 2011). Rosetta generates and refines 3D structural models of the mutated protein and its corresponding wild-type structure, then calculates the energy difference between them. Rosetta employs an energy function in the form of a linear combination of physics-based and knowledge-based contributions. PoPMuSiC is a knowledge-based predictor that uses a statistical energy function trained on a large experimental dataset (Dehouck *et al.* 2011).

Classic machine learning models are by far the most populous category of tools for predicting protein stability (Horne and Shukla 2022). Pancotti *et al.* (2022) thoroughly compared numerous available tools. For example, DDGun (Montanucci *et al.* 2022) is an untrained method that combines three evolutionary sequence-based scores in a linear combination. Its structure-based version, DDGun3D, in addition to the three scores used in DDGun, introduces another term calculated through a statistical potential. Bæk and Kepp introduced simple interpretable linear regression models that achieve accuracy similar to more complex prediction methods (Caldararu *et al.* 2021, Bæk and Kepp 2022). These regression models use only three descriptors: relative solvent accessibility, volume difference, and hydrophobicity difference. PROST (Iqbal *et al.* 2022) is a sequence-based predictor of protein stability upon single-point amino acid change. PROST extracts sequence-based descriptors from predictors such as DDGun and BoostDDG (Lv *et al.* 2020) as well as structure-based descriptors from AlphaFold2 (Jumper *et al.* 2021), and iFeature (Chen *et al.* 2018). The extracted features are used to train an ensemble model based on the XGBoost and extra-trees regressor.

Recently, methods from the last category based on neural network (NN) approaches became popular. This category includes methods such as DeepDDG (Cao *et al.* 2019), ThermoNet (Li *et al.* 2020), SCONES (Samaga *et al.* 2021), ACDC-NN (Benevenuta *et al.* 2021), ACDC-NN-Seq (Pancotti *et al.* 2021), ProS-GNN (Wang *et al.* 2023). Despite the more complex model architecture, this class of methods still does not have a clear advantage over others (Pucci *et al.* 2018, Pak and Ivankov 2022, Pancotti *et al.* 2022).

The performance of a machine learning model largely depends on the training data. Most of the datasets used in studies on protein stability were derived from the ProTherm database (Nikam *et al.* 2021), the largest collection of experimental mutation data. The datasets for model training and testing could be combined in different ways according to experimental conditions, symmetry between stabilizing and destabilizing mutations, and protein sequence similarity. In particular, Pucci *et al.* (2018) have shown the importance of training set symmetry. The authors presented a symmetric test set called Ssym to compare the performance of various models in stabilizing and destabilizing mutations. The results show that most of the models trained on non-symmetric datasets are biased toward destabilizing mutations. Recently, the mega dataset encompassing 800 000 experimentally determined measurements of protein stability changes for miniproteins ranging from 37 to 72 amino acids in length, all conducted in a high-throughput manner was developed (Tsuboyama *et al.* 2023). This dataset is of high interest to train NN models (Pak *et al.* 2023).

In summary, multiple approaches for protein stability prediction have been developed. However, improving the accuracy of the predictions is still of great importance. At the same time, transformers, widely used in many areas of AI since their discovery by Vaswani *et al.* (2017), have only very recently found their way into the field of protein stability prediction (Born and Manica 2023, Jung *et al.* 2023, Zhou *et al.* 2023). In this work, we provide the PROSTATA framework based on the transformer architecture that can be successfully applied to predict changes in protein stability upon single amino acid substitutions.

## 2 Materials and methods

### 2.1 External datasets

In this work, to compare our model with other NNs, we used the original training datasets for the corresponding models where such data were readily available in a unified format. We used Q3421 from STRUM (Quan *et al.* 2016), Q3488 from ThermoNet (Li *et al.* 2020), the widely used S2648 training set provided by Dehouck *et al.* (2011), and additional data from VariBench for ACDC-NN models (Benevenuta *et al.* 2021). Datasets Q3488 and Q3421 were used to assess the effect of a non-symmetric training set on PROSTATA prediction.

The commonly used test sets Ssym (Pucci *et al.* 2018), S669 (Pancotti *et al.* 2022) and protein specific Myoglobin and p53 (Li *et al.* 2020) test sets were chosen to evaluate the models.

### 2.2 Dataset construction

We constructed our own dataset based on relevant sets from the VariBench portal (Nair and Vihinen 2013), including popular training sets such as PoPMuSiC-2.0 (S2648) (Dehouck *et al.* 2011), ThermoNet (Q3214) (Li *et al.* 2020) and VariBench (Nair and Vihinen 2013) (Supplementary Table S1). Data were merged and manually checked.

Since our model does not use experimental conditions as features, we have aggregated samples using a combination of Protein Data Bank (PDB) ID, PDB chain, and mutation code (position and residues in it before and after mutation), from now on referred to as ID. Data were averaged over experimental pH and temperature (T) and pooled in five steps.

1. **Split the data.** All samples were divided into two groups according to whether *pH* and *T* were available (Group I) or not (Group II).
2. **Select core samples.** The samples in Group I with *pH* and *T* closest to the standard values (pH=7 and $T=25^{{}^{\circ}}C$) were selected by ID.
3. **Select additional samples.** From the remaining samples of Group I, for each core sample, we selected the corresponding samples with $pH=pH_{\mathrm{core}}\pm 0.5$ and $T=T_{\mathrm{core}}\pm 10^{{}^{\circ}}C$. Samples with unique IDs to Group II were also selected.
4. **Average** ΔΔG **over mutations.** For each ID of the selected samples, the ΔΔG values were calculated as the mean of the experimental ΔΔG values.
5. **Discard inconsistencies.** To construct the final dataset, the samples with conflicting ΔΔG values (e.g. the opposite signs of ΔΔG values or variance of ΔΔG greater than 5 kJ/mol) were filtered out.

As a result, this dataset comprised 5196 samples (see Supplementary Table S2). The dataset was then expanded by incorporating samples from the mega dataset (Tsuboyama *et al.* 2023), that was processed as outlined in (Pak *et al.* 2023). To maintain diversity and prevent an over-representation of short proteins, we selected 70 samples for each wild-type (WT) protein sequence from the mega dataset, corresponding to the average number of samples for each sequence in the dataset described above. Consequently, the dataset was expanded by 5251 samples.

A reversed mutation was also included for each mutation in the training set to avoid an imbalanced dataset in favor of destabilizing mutations.

The “Hemoglobin” test set was formed by incorporating samples from the PROSTATA dataset that were bound to HEME (HEM), HEME C (HEC), or BILIVERDINE IX ALPHA (BLA) within their corresponding PDB structures.

The “oligomerization” test set was constructed by selecting samples from the PROSTATA dataset that correspond to proteins crystallized in a homo-oligomeric state with over 30% of their residues within a 4.5 Å distance from adjacent subunits.

The “mini_natural” and “mini_denovo” test set were constructed by extracting sets of natural and *de novo* designed miniproteins from the mega dataset (Tsuboyama *et al.* 2023), that shared no homology with proteins from other datasets in this study.

To assess the performance of the PROSTATA framework on the corresponding training and test sets, the training set was refined by excluding homologous sequences using BLAST tool (Camacho *et al.* 2009), which showed higher than 30% sequence identity and hit *E*-value <0.05. This was done to avoid the data leakage between training and test sets leading to inflated performance metrics due to overfitting and to ensure fair comparison with other models. The number of samples in the corresponding training and test set sizes is shown in Supplementary Table S3.

### 2.3 Model architecture

**Figure 2.**
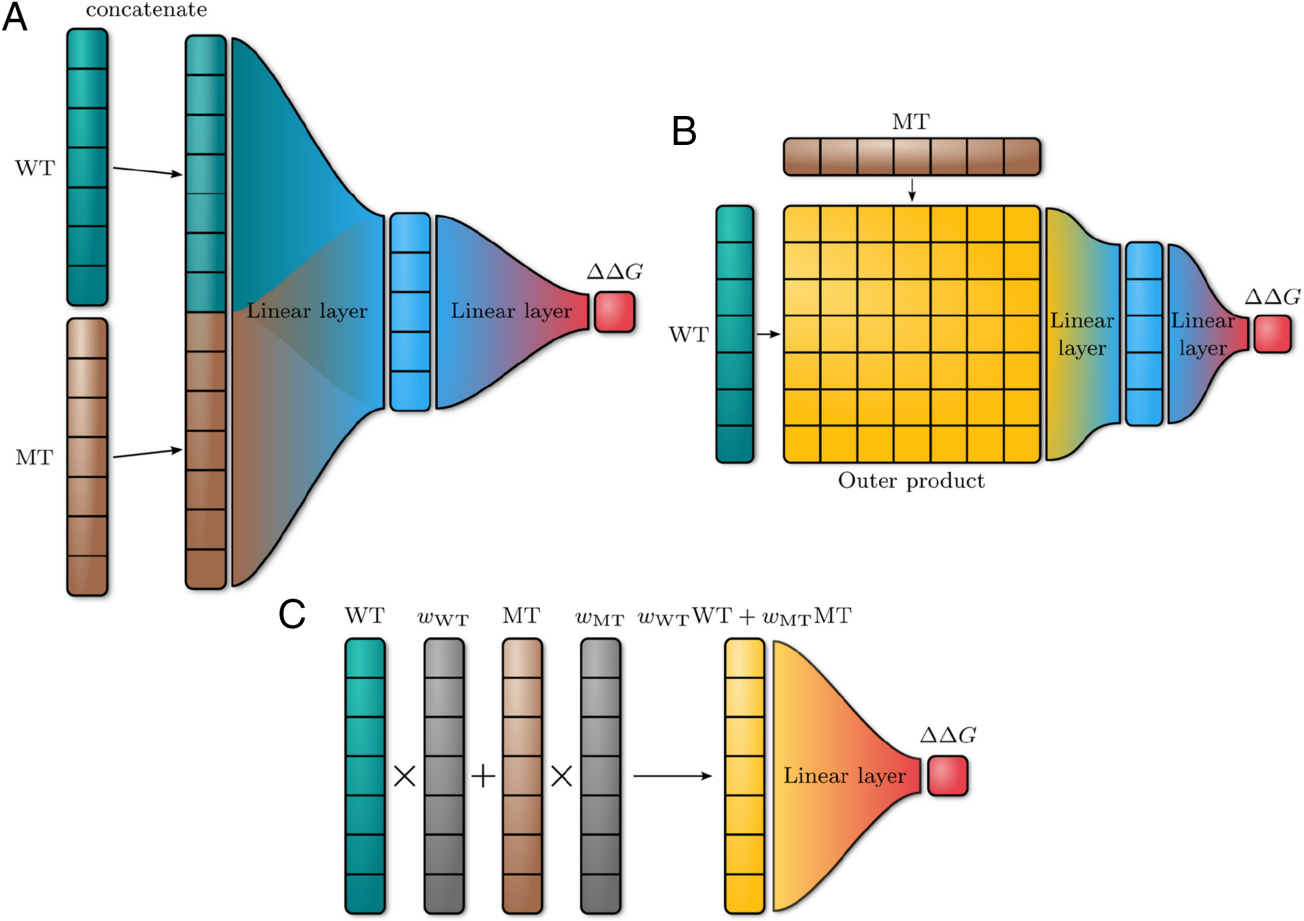
The architecture of the model that uses as input to the NN with one hidden layer (**A**) the concatenation of token embeddings in the mutation position of wild-type (WT) and mutated (MT) protein (**B**) the outer product of token embeddings in the mutation position of wild-type and mutated protein (**C**) the linear combination of wild-type and mutated protein embeddings with vector weights $w_{\text{WT},\text{MT}}$. Multiplication of token embeddings with weight vectors is performed element-wise.

We treat the prediction of the mutation effect on protein stability as a regression task for two sequences, the wild-type and mutated. Using transformer models for this task is a two-step process. First, a model pre-trained on a large corpus of unlabeled data is used to extract the representations of the sequences. Second, the sequence representations of wild-type and mutated proteins are combined into a single representation that is used to predict the target value. Our models consist of a transformer backbone that produces the embeddings for wild-type and mutated proteins and the regression head that combines the embeddings in various ways to predict ΔΔG (Fig. 2). The final predictions were made by averaging the predictions of the five individual models in the ensemble.

### 2.4 Sequence embedding with transformer backbone

Several transformer models pre-trained on unlabeled sequential protein data are available, such as ProtTrans, ProteinBERT, ESM, and ESM-2 (Lin *et al.* 2023). For this work, we have settled on using one of the ESM-2 models as the embedding backbone since these models have outperformed other recent protein language models on downstream tasks (Lin *et al.* 2023). The ESM-2 is a family of models of different sizes with parameter counts ranging from 8 million to 15 billion, with larger models producing better protein representations. For this work, we employ the ESM-2 model with 650 million parameters, as it is the largest model that can be trained on a 32 GB GPU. This model has a hidden layer size of 1280 and produces embeddings of the same size for each residue. Larger models can potentially achieve higher quality at the expense of much longer training and inference times. During sequence embedding, the model calculates representations for each amino acid in the sequence. Additionally, the model calculates representations for special tokens, namely, the classification token (CLS token) inserted at the beginning of each sequence and the END token appended to each sequence. The output of the transformer backbone for each protein sequence of length *N* is a vector of size (N+2)×1280.

### 2.5 Regression head

The second step in the regression pipeline is to combine wild-type (WT) and mutated (MT) embeddings into a joint representation used as input for a linear regression head. A widely used approach in transformer models is using CLS token embeddings for sequence classification. We explored several ways to combine these vectors into a single representation:

- Concatenation of WT and MT embeddings of the mutation position (Fig. 2).
- The outer product of WT and MT embeddings of the mutation position (Fig. 2).
- Linear combination WT and MT embeddings of the mutation position (Fig. 2).
- Linear combination of CLS embeddings (Fig. 2).
- Linear combination of CLS embeddings concatenated with WT and MT embeddings of mutation position (Fig. 2).

### 2.6 Model training and ensembling

All the models were trained with the ADAM optimizer and a batch size of one for three epochs. The learning rate was increased linearly from 0 to 1e−5 during the first 30% of the examples and then linearly decreased to 0 for the remaining examples. We did not freeze the transformer backbone and trained all model weights in an end-to-end manner. The hyperparameters were selected based on (Devlin *et al.* 2018) and prior experiences of the authors with text language models and protein language models (Shashkova *et al.* 2022).

To increase stability and improve the quality of the predictions, we used an ensemble of all five models with different regression heads described previously. The final predictions were made by averaging the predictions of the five individual models in the ensemble.

### 2.7 Model evaluation

We used Pearson correlation (*r*), root mean square error (RMSE), and mean absolute error (MAE) to evaluate PROSTATA performance and compare it with published methods [INPS-Seq (Savojardo *et al.* 2021), ACDC-NN-Seq (Pancotti *et al.* 2021), DDGun (Montanucci *et al.* 2022), PremPS (Chen *et al.* 2020), ThermoNet (Li *et al.* 2020), Rosetta (Kellogg *et al.* 2011), DynaMut (Rodrigues *et al.* 2018, 2021), INPS3D (Savojardo *et al.* 2016), SDM (Worth *et al.* 2011, Pandurangan *et al.* 2017), PoPMuSiC (Dehouck *et al.* 2011), MAESTRO (Laimer *et al.* 2016), DUET (Pires *et al.* 2014)]. These metrics are used in original articles on other methods and in reviews. Therefore, to compare PROSTATA with other publicly available tools on original datasets, we used the performance metrics accordingly with the corresponding articles (Li *et al.* 2020, Benevenuta *et al.* 2021, Wang *et al.* 2023). The performance metrics of various models on the Ssym and the S669 datasets are taken from the work by Pancotti *et al.* (2022).

The sequence profiles for ACDC-NN/ACDC-NN-Seq were obtained by searching for homological sequences with HHblits against the UniRef30 database using default settings (Remmert *et al.* 2011, Mirdita *et al.* 2017).

The ProS-GNN was trained using the provided code at https://github.com/shuyu-wang/ProS-GNN. The training set included the Q3488 dataset and the model was tested on Ssym and Ssym_*r*_ datasets. The model was trained for 400 epochs using Pearson *r* metric on the test set for early stopping, and the non-mutant part of the input PDB files was trimmed by leaving only the mutant and its six adjacent residues.

## 3 Results and discussion

### 3.1 Regression head comparison

We compared the performance of different regression head architectures using 5-fold cross-validation. We used the protein cluster data to build the splits for cross-validation, with each cluster assigned to a single fold. This ensured that the examples in the test set differed from those in the training set for each fold. The results show that none of the models has a clear advantage over the others, while the ensemble of five models has the highest performance (Table 1).

**Table 1.** Results of 5-fold cross-validation for different regression heads.

Embedding	Merge	Pearson *r*	RMSE	MAE
Position	Outer product	0.65	1.65	1.12
Position	Concatenation	0.64	1.64	1.10
CLS	Linear	0.66	1.70	1.44
Position	Linear	0.67	1.60	1.06
CLS + position	Linear	0.67	1.60	1.06

Ensemble		0.69	1.57	1.03

### 3.2 Effects of non-symmetrical datasets

A very desirable quality for a model that predicts the effects of mutations on protein stability is the symmetry of the predictions. In other words, the predicted ΔΔG of a reverse mutation should have the same module as the ΔΔG for the direct mutation and an opposite sign. This quality is not readily achievable for machine learning models [see for a review Fang (2020)]. This symmetry property depends on both the architecture of the model and the dataset which it is trained upon. Machine learning models are now commonly trained on datasets artificially enriched with the effects of reverse mutations.

We examined the effect of the regression head selection on how the model learns the symmetry effects from both symmetric and non-symmetric datasets. For this, we trained our models on the Q3488 and the Q3421 datasets and tested them on the Ssym. The Q3488 dataset contains an equal number of stabilizing and destabilizing mutations, while the Q3421 dataset is heavily biased toward destabilizing mutations. Furthermore, the Q3488 dataset does not contain proteins that are homologous to those found in the Ssym dataset. The results are presented in Table 2.

**Table 2.** Results of models trained on non-symmetric (Q3421) and symmetric (Q3488) sets and tested on the Ssym set.

		Q3421		Q3488	
Embedding	Merge	*r*_dir_	*r*_rev_	*r*_dir_	*r*_rev_
Position	Outer product	0.55	−0.29	0.42	0.44
Position	Concatenation	0.46	−0.39	0.52	0.51
CLS	Linear	0.46	0.47	0.45	0.46
Position	Linear	0.46	0.46	0.45	0.45
CLS + position	Linear	0.42	0.42	0.45	0.45

Ensemble		0.56	0.26	0.51	0.51

The models that use regression heads with the linear merge of wild-type and mutated sequences embeddings can learn the symmetry properly, even when trained on a biased dataset. Models with the outer product and concatenation merging are highly dependent on the balance in the training set and show a negative correlation when presented with the test set with bias reversed from the training set. When provided with a balanced training set, all models are able to perform equally well on direct and reverse mutations.

For further analysis, we decided to use the ensemble of all five modes with different regression heads to ensure the ensemble diversity.

### 3.3 Comparison with other NN models

To disentangle the effect of the architecture versus data, we evaluated the performance of the PROSTATA framework after training on the same training sets as other methods ThermoNet, ACDC-NN, ACDC- NN-Seq, and ProS-GNN. We compared the results on the test sets used by corresponding models. Additionally, we evaluated ACDC-NN/ACDC-NN-Seq on natural and *de novo* design miniproteins extracted from the mega dataset (Tsuboyama *et al.* 2023). To compare the models, we used the Pearson correlation coefficient and RMSE metrics. The metrics of the reviewed models for corresponding training and test sets were taken from the original articles (Li *et al.* 2020, Benevenuta *et al.* 2021, Wang *et al.* 2023) or recalculated if not available.

Among NN-based models, we considered:

- ThermoNet predicts ΔΔG using an ensemble of 3D-CNN (Li *et al.* 2020). ThermoNet treats mutation site environments as multichannel voxel grids parameterized using atom biophysical properties.
- ACDC-NN-Seq is a CNN model that predicts changes in protein stability based on the protein sequence, unlike its predecessor, ACDC-NN, which uses additional 3D structural information (Benevenuta *et al.* 2021). ACDC-NN-Seq takes a sequence profile, containing evolutionary information, together with direct and reverse variations as an input, extracts features using convolution operations, and then feeds them into two differential siamese NNs.
- ProS-GNN (Wang *et al.* 2023) is a deep graph NN that was incorporated into BayeStab (Wang *et al.* 2022), a Bayesian NN that predicts ΔΔG and evaluates the uncertainty of its predictions.

The results of these comparisons are presented in Table 3. These results indicate that PROSTATA demonstrates better or comparable performance to other NNs trained on the same datasets. Importantly, ThermoNet is a framework based on deep 3D-CNNs which uses protein structure as an input and requires features precalculated by Rosetta molecular modeling software (Li *et al.* 2020). ACDC-NN/ACDC-NN-Seq requires evolutionary information to be provided as a sequence profile for the model inference. In contrast, PROSTATA is based on a pre-trained protein language model and only requires a protein sequence and mutation information as input. This provides an advantage in predicting changes in protein stability without the precalculation of additional features. In particular, we compared the performance of the PROSTATA and ACDC-NN/ACDC-NN-Seq on protein stability data of miniproteins extracted from the mega dataset (Tsuboyama *et al.* 2023). This test set was based either on natural (“mini_natural”) and *de novo* (“mini_denovo”) designed miniproteins with limited or no homological proteins available in the UniRef30 database (Mirdita *et al.* 2017). Remarkably, PROSTATA demonstrated equally good performance on both test sets. In the case of ACDC-NN/ACDC-NN-Seq, as expected, performance was lower for *de novo* proteins with no evolutionary information.

**Table 3.** Performance of the NN models on the corresponding training sets and test sets.

		Pearson *r*		RMSE	
Training	Test	Orig^a^	Ours	Orig^a^	Ours
*ThermoNet*					
Q3488	Ssym	0.47	0.51	1.56	1.40
Q3488	Ssym${}_{r}$	0.47	0.51	1.55	1.42
Q3488	p53	0.45	0.59	2.01	1.85
Q3488	Myoglobin	0.38	0.50	1.16	0.98
*ACDC-NN*					
S2648 + Vb${}^{cv}$^b^	Ssym	0.57	0.51	1.45	1.43
S2648 + Vb${}^{cv}$^b^	Ssym${}_{r}$	0.57	0.52	1.45	1.40
S2648 + Vb	p53	0.61	0.66	1.69	1.67
S2648 + Vb	Myoglobin	0.58	0.53	0.89	1.02
S2648 + Vb	mini_natural	0.55	0.63	1.01	0.85
S2648 + Vb	mini_denovo	0.49	0.64	1.25	0.77
*ACDC-NN-Seq*					
S2648 + Vb${}^{cv}$^b^	Ssym	0.55	0.51	1.44	1.43
S2648 + Vb${}^{cv}$^b^	Ssym${}_{r}$	0.55	0.52	1.44	1.40
S2648 + Vb	p53	0.62	0.66	1.62	1.67
S2648 + Vb	Myoglobin	0.56	0.52	0.97	1.01
S2648 + Vb	mini_natural	0.51	0.63	1.05	0.85
S2648 + Vb	mini_denovo	0.45	0.64	1.3	0.77
*ProS-GNN*					
Q3488	Ssym	0.36	0.51	1.69	1.40
Q3488	Ssym${}_{r}$	0.36	0.51	1.69	1.42

a Metrics for reviewed models were taken from original articles or recalculated if not available.

b Model was trained and tested on cross-validation folds (Benevenuta *et al.* 2021).

### 3.4 Evaluation on common test sets

Models that predict the effect of single mutations on protein stability are commonly benchmarked on the Ssym dataset. The greatest challenge of these estimations is the overlap between the training set and the test set, leading to inflated performance metrics (Li *et al.* 2020). Some models such as ThermoNet and SCONES specifically craft their training sets to avoid such an intersection. A recent review of the available tools to predict the effect of single mutations on protein stability (Pancotti *et al.* 2022) introduced a new S669 dataset containing proteins different from commonly used training sets. This dataset allows for a fair comparison of different tools.

We evaluated our model on the S669 test set. Our dataset excluded proteins from the training set with a degree of similarity greater than 30% compared to any protein from the test set. The results are presented in Table 4. PROSTATA achieved the Pearson correlation coefficient of 0.49 for both direct and reverse mutations in the S669 test sets. To compare its performance with other tools, we used metrics obtained from (Pancotti *et al.* 2022). The Pearson correlation coefficient obtained by PROSTATA was higher than that of the sequence-based tools and comparable with the metrics of the structure-based tools.

**Table 4.** Performance of the models on the S669 dataset.^a^

	Direct			Reverse		
Model	*r*	RMSE	MAE	*r*	RMSE	MAE
PROSTATA	0.49	1.45	1.00	0.49	1.45	0.99
INPS-Seq	0.43	1.52	1.09	0.43	1.53	1.10
ACDC-NN-Seq	0.42	1.53	1.08	0.42	1.53	1.08
DDGun	0.41	1.72	1.25	0.38	1.75	1.25
*ACDC-NN*	0.46	1.49	1.05	0.45	1.5	1.06
*DDGun3D*	0.43	1.6	1.11	0.41	1.62	1.14
*PremPS*	0.41	1.5	1.08	0.42	1.49	1.05
*ThermoNet*	0.39	1.62	1.17	0.38	1.66	1.23
*Rosetta*	0.39	2.7	2.08	0.4	2.68	2.02
*Dynamut*	0.41	1.6	1.19	0.34	1.69	1.24
*INPS3D*	0.43	1.5	1.07	0.33	1.77	1.31
*SDM*	0.41	1.67	1.26	0.13	2.16	1.64
*PoPMuSiC*	0.41	1.51	1.09	0.24	2.09	1.64
*MAESTRO*	0.5	1.44	1.06	0.2	2.1	1.66
*DUET*	0.41	1.52	1.1	0.23	2.14	1.68

a Metrics for reviewed models were taken from Pancotti *et al.* (2022). Models in italics are structure-based.

The results on the S669 show that our model improves by a large margin over existing solutions due to a new architecture and the use of a new dataset. Additionally, as some of the existing solutions, the PROSTATA model uses only the amino acid sequence as input without requiring explicit structural, evolutionary, or any other additional features.

### 3.5 Application

PROSTATA was developed to predict the effects of single-point protein substitutions based on amino acid sequences alone. The model’s accuracy should depend primarily on the embeddings derived from the pre-trained protein language model. Protein language models are known to capture structural and evolutionary features (Hie *et al.* 2022, Lin *et al.* 2023), so PROSTATA is expected to be applicable for various protein cases. To evaluate the applicability spectrum of PROSTATA, we measured its performance in a range of difficult cases.

In particular, we tested the predictive capacity of PROSTATA for mutants according to its location within the protein structure, the protein oligomerization state, the solvent solubility, and the presence of small-molecule binding sites.

**Figure 3.**
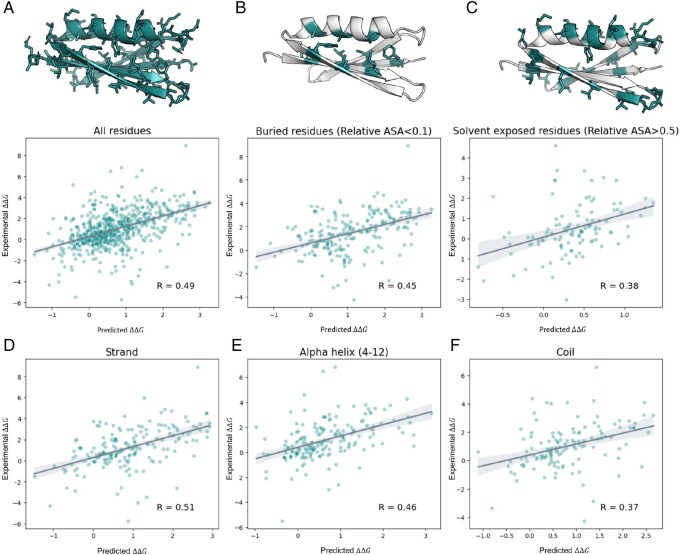
PROSTATA predictions on the S669 test set. (**A–C**) Comparison of PROSTATA performance for buried and solvent-exposed mutant residues. Correlation between predicted and experimental ΔΔG values for all residues (A), buried residues (B), and solvent-exposed residues (C). Regions corresponding to the denoted condition are highlighted on the top. (**D–F**) Comparison of PROSTATA performance for mutants according to the corresponding element of the secondary structure. Correlation between predicted and experimental ΔΔG values for Strand (D), Helix (E), Coil (F). Predictions for both direct and reverse mutations were included. The Pearson correlation coefficient is denoted in the bottom right corner. Representative structures are shown on the top. Relative ASA and secondary structure elements were predicted using the Definition of Secondary Structure of Proteins (DSSP) tool (Kabsch and Sander 1983).

In the first experiment, the mutant positions of the S669 test set were classified according to the location within the protein structure based on the solvent accessibility of the amino acid residues (Fig. 3A–C) and corresponding secondary structure elements (Fig. 3D–F).

We observed that the correlation between the experimental and predicted values was higher for mutant amino acid residues buried in the protein structure than for solvent-exposed residues. This may be due to the fact that changes in stability for solvent-exposed residues, unlike for buried ones, are influenced by the context of what proteins they are exposed to, which is not present in the model input. Beta-strands and alpha-helices (4–12) are the most common secondary structure elements within the experimentally resolved structures. PROSTATA demonstrated the best performance for beta-strand regions, with a slightly lower performance for alpha-helices and coils.

**Figure 4.**
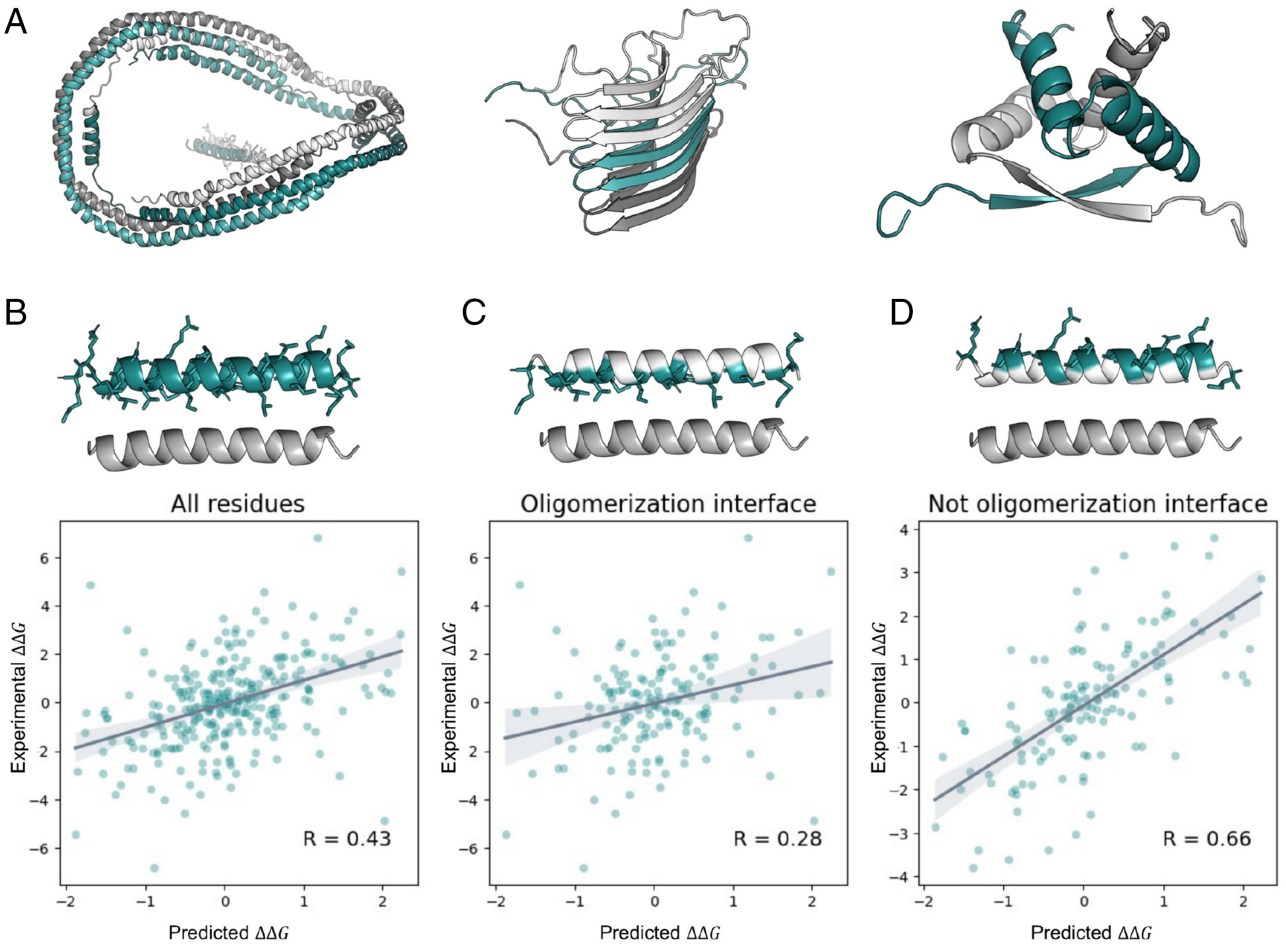
PROSTATA predictions for the test set of oligomeric proteins. (**A**) Representative examples of the test set, including homotrimer (left), amyloid (center), and homodimer (right) structures. (**B–D**) Scatter plots for all mutant residues (B), mutant residues located on oligomerization protein–protein interaction interface (C), and not oligomerization protein–protein interaction interface (D) are shown. Predictions for both direct and reverse mutations were included. The Pearson correlation coefficient is denoted in the bottom right corner. Representative structures are shown on the top. Regions corresponding to the condition are highlighted. Test set included following PDB codes: 1UWO_A, 1R6R_A, 2KJ3_A, 1SCE_A, 1SAK_A, 1ARR_A, 1ZNJ_A, 2A01_A, 2H61_A, 1CDC_B, 1BFM_A, 1ZNJ_B, 1AV1_A, 3MON_B.

Several proteins included in the dataset have a well-packed tertiary fold under biologically relevant conditions only in the oligomeric form. In particular, amyloid peptides are known to be disordered as monomers. Other proteins could be prone to form homodimers or other states of homoligomerization (Fig. 4A). To analyze the performance of PROSTATA in such cases, we developed a test set that includes oligomeric proteins. Proteins were considered oligomeric if at least 30% of residues interacted with other subunits in the experimentally resolved structure within the radii of 4.5 Å. Several representative entities of this test set are shown in Fig. 4A.

**Figure 5.**
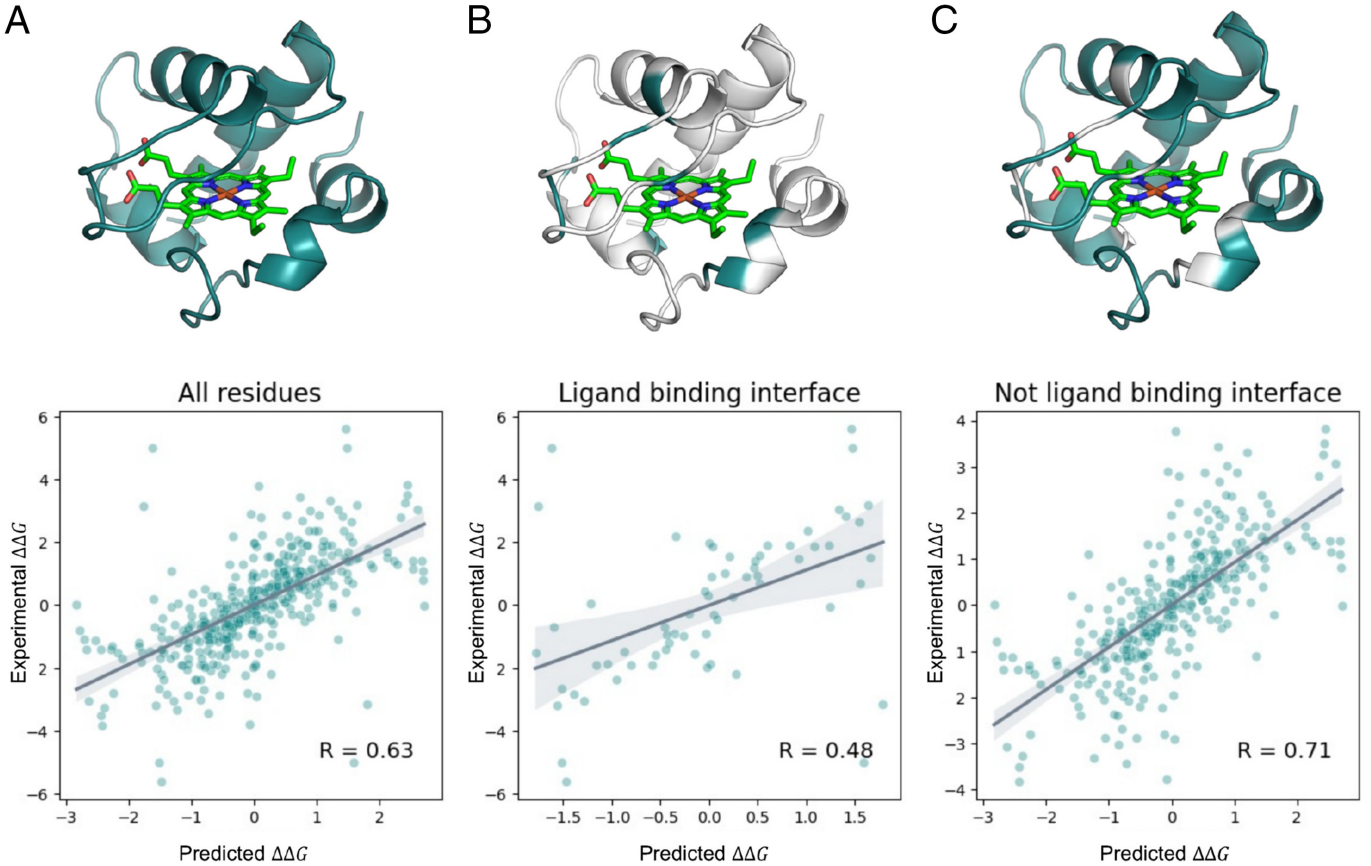
PROSTATA predictions for the class of proteins bound to hemoglobin or hemoglobin-derivatives. (**A**) Correlation between predicted and experimental ΔΔG values for all residues, (**B**) ligand binding interface residues, (**C**) not ligand binding interface residues. Predictions for both direct and reverse mutations were included. The Pearson correlation coefficient is denoted in the bottom right corner. Representative structures are shown on the top. Regions corresponding to the denoted condition are highlighted. Hemoglobin atoms are shown in sticks representation. Test set included following PDB codes: 1C52_A, 1YCC_A, 1CYO_A, 1C2R_A, 1B5M_A, 1AKK_A, 1I5T_A, 1BVC_A, 1YEA_A, 1CYC_A, 451C_A, 1A7V_A.

As expected, the correlation between the experimental and predicted ΔΔG values for the test set was lower than the original. Furthermore, PROSTATA showed low performance only in predicting changes in the ΔΔG values for substitutions located at the protein–protein interaction interface. This is expected since we did not provide any information on protein oligomerization for the model. Therefore, PROSTATA is suitable for monomeric proteins, while for oligomeric proteins, an approach with explicit 3D structures may be more beneficial (Fig. 5).

Other challenging cases might include predicting the effect of mutations in the binding sites of small molecules and cofactors. Cofactor binding usually stabilizes the protein fold, but the location of binding sites is not explicitly encoded. The protein language models might capture the effect implicitly due to evolutionary traits.

To study the performance of PROSTATA, we split the dataset on all hemoglobin or hemoglobin derivative binding proteins and other proteins as a test and training set, respectively. Surprisingly, PROSTATA shows above-average precision in this class of proteins, indicating that the protein language model is able to distinguish well between classes of proteins (Fig. 5). This may be due to many hemoglobin-binding proteins in the UniRef database that were used for training the ESM-2 model (Suzek *et al.* 2015, Lin *et al.* 2023). At the same time, as expected, the overall precision for ligand binding residues was lower than that for other residues.

## 4 Conclusion

In this paper, we used the transfer learning approach to build a predictive model based on combinations of embeddings from the pre-trained protein language model ESM-2. The model PROSTATA is an ensemble of five models with different regression heads. PROSTATA achieves high correlation and low error performance compared to other models being trained on their respective datasets, showing the presented architecture’s benefits. PROSTATA, trained on the dataset presented in the current work, demonstrates the highest performance on the S669 test sets among all other models. Analysis of the performance of our new model on several test sets, including protein classes known to be challenging in ΔΔG prediction, as well as natural and *de novo* designed miniproteins suggests that PROSTATA has acquired broad domain knowledge through transfer learning.

Overall, this work offers a new framework based on large pre-trained protein language models for stability changes prediction.

## Supplementary Material

btad671_Supplementary_Data
